# *N*-Acetyl-*N*-{2-[(*Z*)-2-chloro-3,3,3-tri­fluoro­prop-1-en­yl]phen­yl}acetamide

**DOI:** 10.1107/S1600536809014779

**Published:** 2009-05-14

**Authors:** Jia-Jia Niu, Zhi-Gang Li, Jing-Wei Xu

**Affiliations:** a National Analytical Research Center of Electrochemistry and Spectroscopy, Changchun Institute of Applied Chemistry, Chinese Academy of Sciences, Changchun 130022, People’s Republic of China; b Graduate School of the Chinese Academy of Sciences, Beijing 100039, People’s Republic of China

## Abstract

The title compound, C_13_H_11_ClF_3_NO_2_, adopts a *Z* conformation. Halogen⋯oxygen inter­actions [Cl⋯O = 2.967 (3) Å] in the crystal packing lead to the formation of a dimer joined by two Cl⋯O bonds.

## Related literature

The title compound is an important medical inter­mediate, see: Zhou *et al.* (2009 ▶). For the van der Waals radii of chlorine and oxygen, see: Politzer *et al.* (2007 ▶).
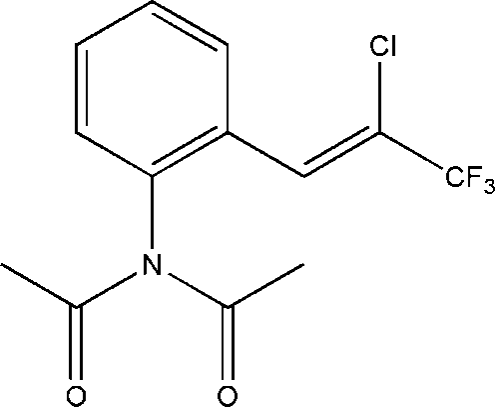


## Experimental

### 

#### Crystal data

C_13_H_11_ClF_3_NO_2_
*M* *_r_* = 305.68
Triclinic, 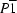

*a* = 8.4408 (19) Å
*b* = 9.385 (2) Å
*c* = 9.455 (2) Å
α = 64.599 (3)°
β = 80.727 (4)°
γ = 89.756 (3)°
*V* = 666.0 (3) Å^3^
*Z* = 2
Mo *K*α radiation
μ = 0.32 mm^−1^
*T* = 293 K
0.13 × 0.13 × 0.07 mm

#### Data collection

Bruker SMART APEX CCD area-detector diffractometer
Absorption correction: multi-scan (*SADABS*; Bruker, 2003 ▶) *T*_min_ = 0.958, *T*_max_ = 0.978
3747 measured reflections
2562 independent reflections
2155 reflections with *I* > 2σ(*I*)
*R*_int_ = 0.010

#### Refinement

*R*[*F* ^2^ > 2σ(*F* ^2^)] = 0.052
*wR*(*F* ^2^) = 0.136
*S* = 1.03
2562 reflections
177 parameters
H-atom parameters constrained
Δρ_max_ = 0.37 e Å^−3^
Δρ_min_ = −0.38 e Å^−3^

### 

Data collection: *SMART* (Bruker, 1998 ▶); cell refinement: *SAINT-Plus* (Bruker, 1998 ▶); data reduction: *SAINT-Plus*; program(s) used to solve structure: *SHELXS97* (Sheldrick, 2008 ▶); program(s) used to refine structure: *SHELXL97* (Sheldrick, 2008 ▶); molecular graphics: *SHELXTL* (Sheldrick, 2008 ▶); software used to prepare material for publication: *SHELXTL*.

## Supplementary Material

Crystal structure: contains datablocks global, I. DOI: 10.1107/S1600536809014779/kj2121sup1.cif

Structure factors: contains datablocks I. DOI: 10.1107/S1600536809014779/kj2121Isup2.hkl

Additional supplementary materials:  crystallographic information; 3D view; checkCIF report

## Figures and Tables

**Fig. 1.**
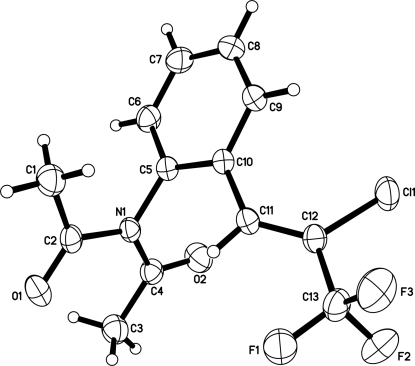
The structure of the title compound, showing the atomic numbering scheme. Displacement ellipsoids are drawn at the 30% probability level.

**Fig. 2.**
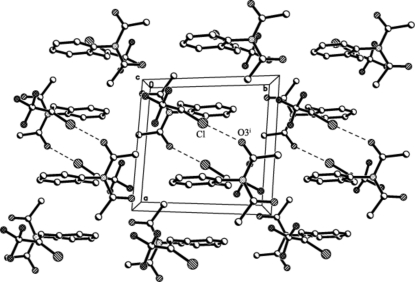
Perspective view of the packing structure of the title compound along the c axis. Dashed lines indicate Cl···O interactions.

## supplementary crystallographic information

### Comment

The title compoud is an important medical intermediate (Zhou *et al.*, 2009). The conformation of the C=C bond (*Z* or *E*) is usually determined by ^1^H and ^19^F NMR. Here we report the crystal structure of title compound to establish the conformation.

The title compound, as shown in Fig. 1, a has *Z* conformation, with the benzene ring and the Cl atom on the same side of C=C bond. In the crystal packing a distance between Cl and O3[-x,-y,1-z) of 2.967 (3) Å is observed, which is obviously shorter than the sum of van der Waals radii of chlorine and oxygen (3.27 Å, Politzer *et al.*,  2007), showing the strong Cl···O interaction indicative of a halogen bond with a nearly linear C12—Cl1···O3[-x,-y,1-z] angle of 173.5 (4)°. Two monomers, related by a centre of symmetry, are linked into a dimer by two Cl···O halogen bonds (Fig. 2). There are no distinct interactions between the dimers in the crystal packing.

### Experimental

*N*-(2-formylphenyl)acetamide (1 mmol) and zinc powder (5 mmol) were added to DMF (5 ml, distilled from CaH_2_), the flash was then evacuated and backfilled with argon (3 cycles). Acetic anhydride (3 mmol) was added by syringe at room temperature, and then 3 mmol 1,1,1-trichloro-2,2,2-trifluoro-ethane was added to the reaction mixture slowly in 10 minutes. The reaction mixture was stirred at room temperature for 3 h. The mixture was partitioned between ethyl acetate and water. The organic layer was separated, and the aqueous layer was extracted with ethyl acetate. The combined organic layers were washed with brine, dried over Na_2_SO_4_, and concentrated *in vacuo*. The residual oil was loaded on a silica gel column and eluted with ethyl acetate/petroleum ether (1/9) to afford the product (55%). The purified product was recrystallized from petroleum ether to get colorless block crystals

### Refinement

H atoms were placed geometrically and refined with fixed individual displacement parameters [*U*_iso_(H) = 1.2*U*_eq_(C,*N*)], using a riding model, with C—H distances of 0.93 Å for C*sp*^2^ [*U*_iso_(H) = 1.2*U*_eq_(C)] and 0.96 Å for methyl C [*U*_iso_(H) = 1.5*U*_eq_(C)].

### Crystal data

**Table:**

C_13_H_11_ClF_3_NO_2_	*Z* = 2
*M**_r_* = 305.68	*F*(000) = 312
Triclinic, *P*1	*D*_x_ = 1.524 Mg m^−^^3^
Hall symbol: -P 1	Mo *K*α radiation, λ = 0.71073 Å
*a* = 8.4408 (19) Å	Cell parameters from 1359 reflections
*b* = 9.385 (2) Å	θ = 2.3–24.8°
*c* = 9.455 (2) Å	µ = 0.32 mm^−^^1^
α = 64.599 (3)°	*T* = 293 K
β = 80.727 (4)°	Block, colorless
γ = 89.756 (3)°	0.13 × 0.13 × 0.07 mm
*V* = 666.0 (3) Å^3^	

### Data collection

**Table:**

Bruker APEX CCD area-detector diffractometer	2562 independent reflections
Radiation source: fine-focus sealed tube	2155 reflections with *I* > 2σ(*I*)
graphite	*R*_int_ = 0.010
φ and ω scans	θ_max_ = 26.0°, θ_min_ = 2.4°
Absorption correction: multi-scan (*SAINT-Plus*; Bruker, 2003)	*h* = −10→7
*T*_min_ = 0.958, *T*_max_ = 0.978	*k* = −11→10
3747 measured reflections	*l* = −11→11

### Refinement

**Table:**

Refinement on *F*^2^	Primary atom site location: structure-invariant direct methods
Least-squares matrix: full	Secondary atom site location: difference Fourier map
*R*[*F*^2^ > 2σ(*F*^2^)] = 0.052	Hydrogen site location: inferred from neighbouring sites
*wR*(*F*^2^) = 0.136	H-atom parameters constrained
*S* = 1.03	*w* = 1/[σ^2^(*F*_o_^2^) + (0.06*P*)^2^ + 0.4882*P*] where *P* = (*F*_o_^2^ + 2*F*_c_^2^)/3
2562 reflections	(Δ/σ)_max_ < 0.001
177 parameters	Δρ_max_ = 0.37 e Å^−^^3^
0 restraints	Δρ_min_ = −0.38 e Å^−^^3^

### Special details

**Table:**

Geometry. All e.s.d.'s (except the e.s.d. in the dihedral angle between two l.s. planes) are estimated using the full covariance matrix. The cell e.s.d.'s are taken into account individually in the estimation of e.s.d.'s in distances, angles and torsion angles; correlations between e.s.d.'s in cell parameters are only used when they are defined by crystal symmetry. An approximate (isotropic) treatment of cell e.s.d.'s is used for estimating e.s.d.'s involving l.s. planes.
Refinement. Refinement of *F*^2^ against ALL reflections. The weighted *R*-factor *wR* and goodness of fit *S* are based on *F*^2^, conventional *R*-factors *R* are based on *F*, with *F* set to zero for negative *F*^2^. The threshold expression of *F*^2^ > σ(*F*^2^) is used only for calculating *R*-factors(gt) *etc*. and is not relevant to the choice of reflections for refinement. *R*-factors based on *F*^2^ are statistically about twice as large as those based on *F*, and *R*- factors based on ALL data will be even larger.

### Fractional atomic coordinates and isotropic or equivalent isotropic displacement parameters (Å^2^)

**Table:**

	*x*	*y*	*z*	*U*_iso_*/*U*_eq_	
Cl1	0.34597 (9)	0.48032 (9)	1.17904 (9)	0.0624 (3)	
F1	0.1487 (3)	0.0570 (2)	1.3535 (2)	0.0876 (7)	
F2	0.3488 (2)	0.1468 (2)	1.4086 (2)	0.0839 (6)	
F3	0.1179 (3)	0.2263 (3)	1.4482 (2)	0.0849 (6)	
N1	0.2505 (2)	0.2173 (2)	0.8013 (2)	0.0395 (5)	
O1	0.1014 (3)	−0.0027 (2)	0.8367 (3)	0.0615 (5)	
O2	0.5147 (2)	0.2397 (3)	0.8026 (3)	0.0669 (6)	
C1	−0.0380 (4)	0.2291 (4)	0.7833 (4)	0.0625 (6)	
H1A	−0.1288	0.1624	0.7927	0.094*	
H1B	−0.0184	0.3158	0.6790	0.094*	
H1C	−0.0600	0.2692	0.8617	0.094*	
C2	0.1066 (3)	0.1358 (3)	0.8087 (3)	0.0439 (6)	
C3	0.4252 (4)	−0.0064 (4)	0.8221 (4)	0.0625 (6)	
H3A	0.5363	−0.0280	0.8237	0.094*	
H3B	0.3932	−0.0141	0.7325	0.094*	
H3C	0.3602	−0.0820	0.9185	0.094*	
C4	0.4031 (3)	0.1564 (3)	0.8091 (3)	0.0462 (6)	
C5	0.2485 (3)	0.3838 (3)	0.7692 (3)	0.0387 (5)	
C6	0.2754 (3)	0.4964 (3)	0.6127 (3)	0.0501 (6)	
H6	0.2997	0.4655	0.5310	0.060*	
C7	0.2665 (4)	0.6545 (3)	0.5773 (3)	0.0585 (7)	
H7	0.2850	0.7301	0.4720	0.070*	
C8	0.2300 (4)	0.6998 (3)	0.6988 (4)	0.0564 (7)	
H8	0.2196	0.8059	0.6753	0.068*	
C9	0.2089 (3)	0.5884 (3)	0.8548 (3)	0.0483 (6)	
H9	0.1863	0.6208	0.9356	0.058*	
C10	0.2205 (3)	0.4277 (3)	0.8945 (3)	0.0376 (5)	
C11	0.2003 (3)	0.3065 (3)	1.0601 (3)	0.0401 (5)	
H11	0.1495	0.2099	1.0813	0.048*	
C12	0.2460 (3)	0.3182 (3)	1.1831 (3)	0.0419 (5)	
C13	0.2142 (4)	0.1867 (4)	1.3472 (3)	0.0559 (7)	

### Atomic displacement parameters (Å^2^)

**Table:**

	*U*^11^	*U*^22^	*U*^33^	*U*^12^	*U*^13^	*U*^23^
Cl1	0.0723 (5)	0.0633 (5)	0.0603 (5)	−0.0114 (3)	−0.0172 (3)	−0.0328 (4)
F1	0.1321 (18)	0.0637 (11)	0.0503 (10)	−0.0312 (11)	−0.0168 (11)	−0.0086 (9)
F2	0.0850 (13)	0.0953 (15)	0.0632 (12)	0.0159 (11)	−0.0323 (10)	−0.0202 (11)
F3	0.0907 (14)	0.1127 (16)	0.0463 (10)	0.0056 (12)	0.0090 (9)	−0.0369 (10)
N1	0.0450 (11)	0.0410 (10)	0.0386 (11)	0.0010 (8)	−0.0083 (8)	−0.0226 (9)
O1	0.0679 (12)	0.0511 (11)	0.0724 (14)	−0.0081 (9)	−0.0115 (10)	−0.0334 (10)
O2	0.0480 (11)	0.0663 (13)	0.0952 (17)	0.0048 (9)	−0.0180 (11)	−0.0414 (12)
C1	0.0555 (12)	0.0646 (13)	0.0786 (15)	0.0087 (10)	−0.0172 (10)	−0.0398 (12)
C2	0.0502 (14)	0.0494 (14)	0.0369 (13)	−0.0043 (11)	−0.0062 (10)	−0.0235 (11)
C3	0.0555 (12)	0.0646 (13)	0.0786 (15)	0.0087 (10)	−0.0172 (10)	−0.0398 (12)
C4	0.0487 (14)	0.0510 (14)	0.0452 (14)	0.0062 (11)	−0.0096 (11)	−0.0263 (12)
C5	0.0398 (12)	0.0399 (12)	0.0389 (12)	0.0010 (9)	−0.0073 (10)	−0.0193 (10)
C6	0.0584 (16)	0.0553 (15)	0.0371 (13)	0.0008 (12)	−0.0074 (11)	−0.0208 (12)
C7	0.0726 (19)	0.0469 (15)	0.0438 (15)	−0.0042 (13)	−0.0144 (13)	−0.0067 (12)
C8	0.0687 (18)	0.0388 (14)	0.0620 (18)	0.0044 (12)	−0.0221 (14)	−0.0184 (13)
C9	0.0547 (15)	0.0457 (14)	0.0532 (15)	0.0074 (11)	−0.0149 (12)	−0.0277 (12)
C10	0.0375 (12)	0.0399 (12)	0.0391 (12)	0.0006 (9)	−0.0080 (9)	−0.0202 (10)
C11	0.0433 (12)	0.0404 (12)	0.0404 (13)	−0.0009 (10)	−0.0045 (10)	−0.0219 (11)
C12	0.0416 (12)	0.0485 (13)	0.0402 (13)	0.0009 (10)	−0.0040 (10)	−0.0247 (11)
C13	0.0649 (17)	0.0642 (18)	0.0407 (14)	−0.0006 (14)	−0.0095 (13)	−0.0246 (13)

### Geometric parameters (Å, °)

**Table:**

Cl1—C12	1.726 (2)	C3—H3C	0.9600
F1—C13	1.312 (3)	C5—C6	1.382 (3)
F2—C13	1.338 (3)	C5—C10	1.399 (3)
F3—C13	1.328 (3)	C6—C7	1.378 (4)
N1—C4	1.407 (3)	C6—H6	0.9300
N1—C2	1.414 (3)	C7—C8	1.378 (4)
N1—C5	1.458 (3)	C7—H7	0.9300
O1—C2	1.209 (3)	C8—C9	1.377 (4)
O2—C4	1.204 (3)	C8—H8	0.9300
C1—C2	1.489 (4)	C9—C10	1.396 (3)
C1—H1A	0.9600	C9—H9	0.9300
C1—H1B	0.9600	C10—C11	1.471 (3)
C1—H1C	0.9600	C11—C12	1.329 (3)
C3—C4	1.493 (4)	C11—H11	0.9300
C3—H3A	0.9600	C12—C13	1.493 (4)
C3—H3B	0.9600		
			
C4—N1—C2	125.8 (2)	C5—C6—H6	119.9
C4—N1—C5	115.01 (19)	C8—C7—C6	119.7 (3)
C2—N1—C5	119.0 (2)	C8—C7—H7	120.2
C2—C1—H1A	109.5	C6—C7—H7	120.2
C2—C1—H1B	109.5	C9—C8—C7	120.1 (2)
H1A—C1—H1B	109.5	C9—C8—H8	119.9
C2—C1—H1C	109.5	C7—C8—H8	119.9
H1A—C1—H1C	109.5	C8—C9—C10	121.5 (2)
H1B—C1—H1C	109.5	C8—C9—H9	119.3
O1—C2—N1	121.5 (2)	C10—C9—H9	119.3
O1—C2—C1	122.1 (2)	C9—C10—C5	117.2 (2)
N1—C2—C1	116.3 (2)	C9—C10—C11	122.6 (2)
C4—C3—H3A	109.5	C5—C10—C11	120.2 (2)
C4—C3—H3B	109.5	C12—C11—C10	127.9 (2)
H3A—C3—H3B	109.5	C12—C11—H11	116.1
C4—C3—H3C	109.5	C10—C11—H11	116.1
H3A—C3—H3C	109.5	C11—C12—C13	122.5 (2)
H3B—C3—H3C	109.5	C11—C12—Cl1	126.5 (2)
O2—C4—N1	118.3 (2)	C13—C12—Cl1	111.00 (18)
O2—C4—C3	121.3 (2)	F1—C13—F3	107.5 (2)
N1—C4—C3	120.4 (2)	F1—C13—F2	106.4 (3)
C6—C5—C10	121.0 (2)	F3—C13—F2	105.2 (2)
C6—C5—N1	118.5 (2)	F1—C13—C12	113.0 (2)
C10—C5—N1	120.5 (2)	F3—C13—C12	112.0 (2)
C7—C6—C5	120.3 (2)	F2—C13—C12	112.2 (2)
C7—C6—H6	119.9		
